# Textile-Based Weft Knitted Strain Sensors: Effect of Fabric Parameters on Sensor Properties

**DOI:** 10.3390/s130811114

**Published:** 2013-08-21

**Authors:** Ozgur Atalay, William Richard Kennon, Muhammad Dawood Husain

**Affiliations:** 1 School of Materials, University of Manchester, Manchester, M60 1QD, UK; E-Mail: Richard.kennon@manchester.ac.uk; 2 Department of Textile Engineering, NED University of Engineering and Technology, University Road 75270, Karachi, Pakistan

**Keywords:** strain sensor, yarn input tension, elastomeric yarn, conductive yarn, contact pressure, contact area, conductive textiles

## Abstract

The design and development of textile-based strain sensors has been a focus of research and many investigators have studied this subject. This paper presents a new textile-based strain sensor design and shows the effect of base fabric parameters on its sensing properties. Sensing fabric could be used to measure articulations of the human body in the real environment. The strain sensing fabric was produced by using electronic flat-bed knitting technology; the base fabric was produced with elastomeric yarns in an interlock arrangement and a conductive yarn was embedded in this substrate to create a series of single loop structures. Experimental results show that there is a strong relationship between base fabric parameters and sensor properties.

## 1. Introduction

Electro-textiles can be defined as textiles with unobtrusively built-in electronic and photonic functions [1]. They are mostly used for electromagnetic shielding, anti-static and heating purposes, and also for soft circuits: electric circuits or sensors made out of a combination of special fabrics, threads, yarns and electronic components [2]. Electrical functions can be embedded in textiles by using weaving, knitting and embroidery or nonwoven production techniques. The integration of electronic properties directly into the clothing environment carries some advantages such as increased comfort, mobility, usability and aesthetic properties. However, there are some challenges to be addressed. Yarns that are used for making cloth should be fine and elastic in order to ensure the wearer's comfort. The fibres have to be able to withstand handling and fabrics should have low mechanical resistance to bending and shearing which means they can be easily deformed and draped [3].

The creation of textile-based strain sensors has attracted researchers' attention so many investigators have studied this area and numerous different kinds of technique have been used in order to create strain sensing structures. These sensors have been used to measure human body movements or respiratory activity [4–10]. De Rossi *et al.* [11,12] created strain sensing fabrics by coating Lycra/cotton fabrics with polypyrrole and carbon loaded rubbers. Polypyrrole-coated fabrics showed an average gauge factor of about −13. These strain sensing fabrics exhibited a strong variation of strain-resistance with time and they showed a high response time to applied mechanical stimulus. Fabrics coated with carbon loaded rubber had a gauge factor of approximately 2.5 and fabric sensors made with this type of material showed good strain sensing properties between 1% and 13% strain. Xue *et al.* [13] also created strain sensing structures by coating nylon 6 and polyurethane fibres with polypyrrole. According to this research, polypyrrole-coated nylon 6 fibres showed good sensing performance, whereas polypyrrole-coated polyurethane fibres did not produce promising results as a strain sensing structure. Also, Mattmann *et al.* [14] created a strain sensor by using a thermoplastic elastomer and carbon particles and they were able to recognize upper body postures with an accuracy of 97%.

Another method for the creation of textile based strain sensing structures is the embedding of conductive yarns into knitted or woven structures. As a distinction from the coated sensors, they offer an integration of the sensing part during the manufacturing stage of the fabric. Thus, this approach reduces the production stage to one step. Since woven fabrics are generally characterised by their dimensional stability, poor skin contact and limited elastic recovery, knitted structures are more suitable for strain sensor applications as it is easier to create flexible structures which fit closely against the human body. Zhang *et al.* [15,16] created knitted strain sensors by using stainless steel yarns and carbon yarns and identified that the contacting electrical resistance between overlapped fibres is the primary factor in the sensing mechanism. They also considered electrically conductive fabrics as pure resistive networks and found a solution for the plain fabric circuit network. Yang *et al.* [17] modelled 1 × 1 conductive rib fabrics as resistive networks and found a relationship between extension and the equivalent resistance both experimentally and theoretically. Li *et al.* [18] also investigated the relationship between the electrical resistance and textile force which includes the length related resistance of conductive yarns and the contact resistance of two overlapped yarns. However, the effects of base fabric knitting parameters on sensor characteristics were not studied in the earlier research.

The primary objective of this new study was to develop fabric-based strain sensors using the knitting route. Elastomeric yarns with different linear yarn density have been used to create interlock based structures, because the interlock structure has the highest dimensional stability among the basic weft knitted structures. Thus, this characteristic enables the creation of more reliable sensors in terms of repeatability. Silver coated polymeric yarn was used as a sensing element and this was knitted over the interlock base structure as a series of single loops of the fabric which were arranged to help reduce the conductive yarn structural deformation during long term force loading. Different elastomeric yarn input tensions were applied to produce interlock structures with different fabric compactness in order to investigate the effect of contact pressure on the electrical resistance as well as sensor characteristics. The following section describes the electro-mechanic theory and production of knitted strain sensors followed by the testing method for the electromechanical properties of the sensors. The third part reports the results obtained from the experimental procedure and discussion of the electro-mechanical properties of the sensor.

## 2. Materials and Methods

### 2.1. Production of Knitted Strain Sensing Fabrics

A single design of knitted strain-sensing fabric was devised and three different variations of the basic knitted sensor were created using 800 decitex (the mass in gram of 10,000 m of yarn), 570 decitex double covered elastomeric yarns and silver coated nylon yarn. The three groups of sensing structures, each with different characteristics, were manufactured with a Shima Seiki SES 122-S ten gauge computerised flat-bed knitting machine by varying the elastomeric yarn input tension and linear yarn density. Table 1 presents the manufacturing parameters of the knitted strain-sensing fabrics.

**Figure 1.**
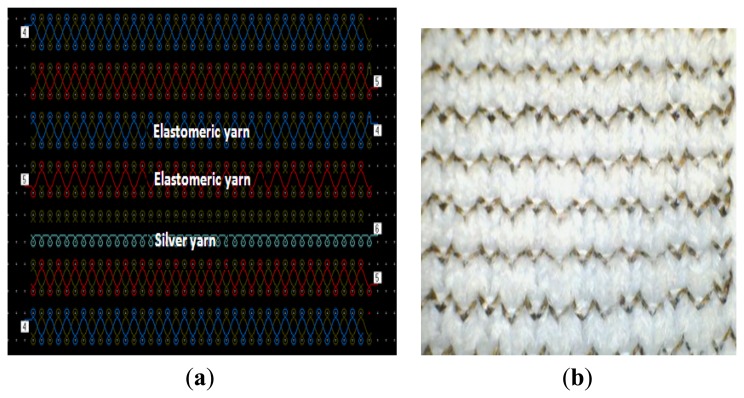
(**a**) Needle notation for knittedstrain-sensing fabric shows arrangement of elastomeric and silver yarn in the structure; (**b**) Technical face of knitted sampleshows conductive loops in the structure.

**Figure 2.**
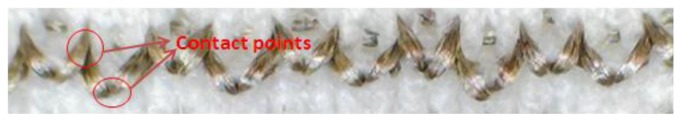
Magnified image of knitted sensor showing a conductive course.

**Figure 3.**
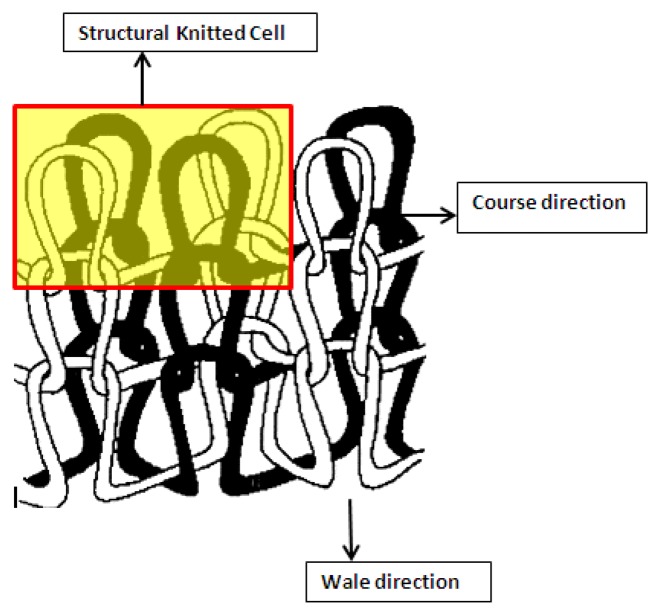
Schematic diagram of Interlock fabric showing a structural knitted cell.

At the technical face of the fabric, silver plated nylon yarn was used to create conductive loops; it has 235 decitex fineness and 200 Ω/m linear resistance. The three groups of strain sensing fabrics were manufactured using an interlock arrangement and the conductive yarn was embedded into this interlock structure in a series of single loops. It should also be noted that the same amount of silver yarn was used for each group during the manufacturing process, so the length of conductive yarn was kept equal for each sample. The needle notation, carrier locations and front side photograph of the knitted samples are shown in Figure 1 and a magnified image of a sample is shown in Figure 2. Five specimens were prepared for each group. Samples belonging to the first two groups were produced using 800 decitex elastomeric yarns with an accurately controlled run-in tension of 0.125 cN/Tex. The second batch of samples was knitted with an accurately-controlled run-in tension of 0.062 cN/Tex on the elastomeric yarn. The Third group of samples was produced using 570 decitex elastomeric yarns with run-in tension of 0.125 cN/Tex during knitting. Thus, samples with three different tightness factors were created and this varied the compactness of the knitted structures. The tightness factor in the metric system may be expressed as $\sqrt{Tex}/1$, where the yarn linear density is in Tex and the loop length (l) is expressed in mm [19]. In this study, Knapton's [20] structural knitted cell (SKC) concept was adopted for calculation of interlock fabric loop length. According to this concept, the effective loop length is the length of yarn in one SKC which consists of four single loops. The schematic diagram in Figure 3 shows one structural knitted cell in the interlock fabric. Compactness is an important fabric property which affects fabric properties including dimensional stability, strength, drape, handle and shrinkage. Normally, structures with high a tightness factor have higher wale and course stitch density values. Since course and wale spacing decreases, higher contact pressure occurs between adjacent courses and wales and this study will reveal the effect of the tightness factor on fabric electrical resistance and the sensing characteristics.

### 2.2. Electro-Mechanical Theory

#### 2.2.1. Gauge Factor

In strain sensors, the gauge factor (GF) is an important parameter and it gives information about the sensitivity of the sensor. The GF is calculated as follows:

(1) $$\text{GF}=\frac{\frac{\Delta \text{R}}{\text{R}}}{\varepsilon }$$

where:

ΔR = the change in the resistance;
R = the initial resistance (the resistance before extension);
ε = the strain value.

#### 2.2.2. Structural Design

**Figure 4.**
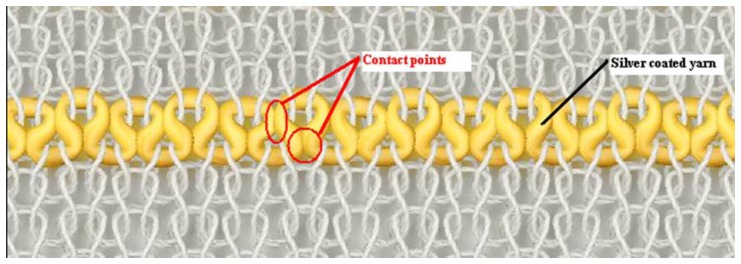
Schematic diagram of sensor design showing the geometry of the conductive yarn.

As may be seen from Figure 4, due to the usage of elastomeric yarn in the structure, conductive yarn loops make contact with adjacent loops at their heads and limbs also at their sinker loops which are pressed together. In addition to this, conductive loops are located within the interlock structure in the form of a zigzag arrangement in which they are alternately located in a higher or lower position relative to each other. This is due to the modification of the conductive plain loops which are used in the interlock structure. This feature improves contact area significantly and enables sensor measure up high strain levels. It is not clear from the needle notation of the knitted sensor shown Figure 1 that adjacent loops of conductive yarns can be designed to touch each other. Hence, this type of sensor derives predominantly from practical knitting experience. These are functions of the design of the knitted sensor and are enhanced by the incorporation of elastomeric yarn and interlock structure.

#### 2.2.3. Contact Theory

According to Holm's [21] contact theory:

(2) $${\text{R}}_{\text{c}}=\frac{\rho }{2}\sqrt{\frac{\pi .H}{nP}}$$

where:

R_c_ = contact resistance;
ρ = electrical resistivity;
H = material hardness;
n = number of contact points;
P = contact pressure.

From Equation (2), it can be see that the electrical resistivity and material hardness are constant for a given material, but the number of contact points and the contact pressure are variable depending on the sensor design. Thus, higher contact pressure and increased number of contact points between conductive parts lower the contact resistance. In this sensor design, contact pressure between the conductive loops has a maximum value before extending the fabric, but during the force loading stage, uniaxial tensile force reduces the level of contact between the conductive loops. Hence, contact pressure between conductive loops lessens depending on the level of applied strain, so the overall electrical resistance of the proposed sensor increases with strain. The three groups of sensors with different compactness have been produced In order to study the effect of contact pressure between the conductive loops and to characterise the sensor behaviour.

### 2.3. Test Procedure for Knitted Strain Sensors

In order to calculate the GF and to investigate the sensor characteristics of the knitted strain sensing fabrics, electro-mechanical measurements of the various samples were performed under multi-cyclic tensile stress using a Zwick/Roell BTC-FR2.5TS.D09 tensile testing machine to apply repeated mechanical extension and deformation. The change of resistance was measured simultaneously with applied strain by the tensile tester in combination with a Wheatstone bridge arrangement. Experimental data was recorded using the Testexpert software. The samples were subjected to levels of up to 40% extension in the course direction and the change of electrical resistance was recorded over the time. The extension level of 40% was chosen to mirror typical human body extensions, as the proposed sensor can be used for monitoring human body movements. The tensile testing machine has a fixed and a moveable crosshead which may be driven at a range of speeds. In this research, samples were tested with a constant rate of extension of 120 mm/min with a full test comprising 20 repeats. In addition to the multi-cyclic tensile test, two further different tests were performed. Firstly, fabric samples were subjected to conditioning extension with a two minute dwell time at 40% strain and a two minute dwell time at 0% in order to study the relaxation behaviour of the sensor. Thereafter, each knitted strain sensing fabric was extended up to the 40% strain level at 120 mm/min speed with 1,000 repeats so as to investigate the effect of long term cycling.

## 3. Results and Discussion

### 3.1. Comparison of Average Electrical Resistance and Average Base Fabric Parameters

The initial electrical resistance of the knitted strain sensors was measured before any external applying tensile forces in order to see the effect of base fabric knitting parameters on the electrical resistance of five samples. Table 2 shows the average electrical resistance values and the base fabric knitting parameter values.

When the first two groups of samples are compared, samples belonging to group 1 demonstrate a lower average electrical resistance value. A number of aspects of the work have been considered which may help to explain this situation. Samples belonging to group 1 have higher average wale density, so more pressure is exerted on the touching points of the conductive yarn loops. According to Holm's contact resistance theory, when the contact pressure and number of contact points increase, then the contact resistance decreases. In addition to this, more contact points are generated by higher wale density and this also causes the resistance values to be reduced. As seen in Table 2, group 3 samples were produced by applying same elastomeric yarn input tension per Tex as those in group 1. Since lower linear yarn density was used, group 3 samples have highest wale density as well as the highest course density. In the light of this information, group 3 samples were expected to have the lowest resistance values. However, in this case the elastomeric linear yarn density affected the measurements significantly. As some of the conductive yarn loop contact points are located between the wales of interlock fabric, the finer elastomeric yarn which was used for group 3 created fewer contact areas in comparison to using thicker yarn. Thus, the average electrical resistance of the group 3 samples was higher than group 1 samples. However, since the amount of silver yarn was kept constant, the conductive loops created an enhanced ridge effect at the lowest stitch length values as conductive yarn loops located on the interlock stitch.

### 3.2. Sensor Characterisation

**Figure 5.**
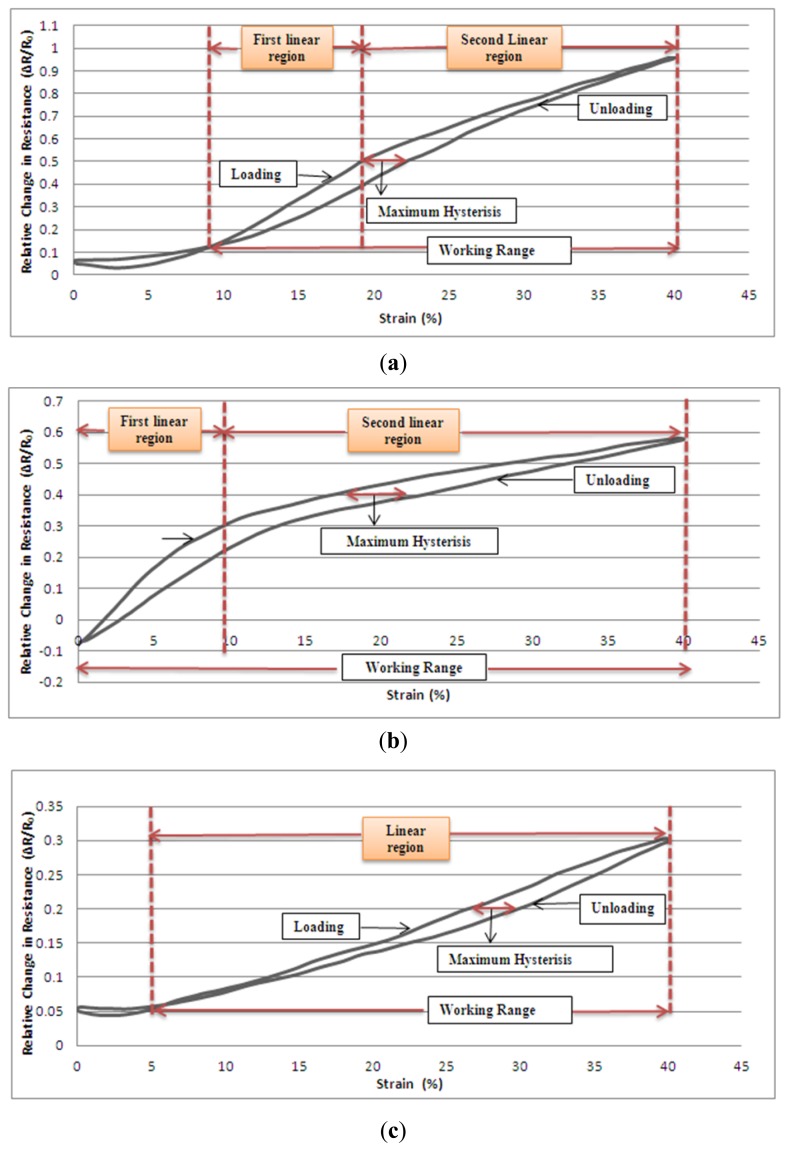
Relative change in resistance-strain graphs of three groups. (**a**) Group 1; (**b**) Group 2; (**c**) Group 3.

The graphs in Figure 5 show the relative change in resistance *versus* strain for the three groups of samples whilst they are being subjected to cyclic tensile testing. The samples were cycled between 0% and 40% strain at a speed of 120 mm/min, with 20 repeats and there were no dwell times at either the lowest or the highest strain levels. 20 sets of measurement were averaged for plotting each graph. It can be seen in the graphics of Figure 5 that there are in fact two hysteresis loops described by the curves and these extend from 0% to around 8% strain and from 8% to 40% strain respectively for group 1 sensors; from 0% to around 5% strain and from 5% to 40% strain for group 3 sensors. The reason for this behaviour is that the applied strain creates textile deformation over the fabric to such an extent that group 1 and 3 sensors starts to experience time-depended recovery. Thus, during the cyclic tests, this level of deformation stretches the fabric to its elastic recovery limit and buckling is apparent in the samples when the strain is released. It does not appear that has been inflicted on the sensors, but recovery takes an extended period of time. When loading is applied to a buckled sample, the fabric is initially pulled flat and this causes the touching points of the conductive knitted loops to make enhanced contact with each other so there is a slight decrease in resistance in the very early stage of straining a distorted sample. This phenomenon occurs only up to the 2.8% level of strain for group 1 sensors and 2.4% level of strain for group 3 sensors. Hence, the working range of sensors can be considered as being between the finishing strain values of first hysteresis loops and the 40% strain limit. Also, the maximum hysteresis values of sensors are 3%, 5.8% and 3.4% for group 1, group 2 and group 3 sensors, respectively. As seen from the graphs, the three groups of sensors demonstrate different behaviour and different gauge factors with applied strain. When the first two groups which are produced with the same linear yarn density were compared, group 1 showed more linear response over the 40% strain range. Actually both of the sensors can be characterised by two linear regions at their working range. Group 1 sensors have an initial linear region up to 19% strain, then a second linear region between 19% and 40% strain. The gauge factors values of sensors have been calculated to be close 3.75 for strains below 19% then the gauge factor falls to 2.16 for strains between19% and 40%. Group 2 sensors have their first linear region up to 9% strain and their second linear region between 9% and 40% strain and the gauge factor values are approximately 4.3 and 0.9 respectively for the given regions. As group 1 samples are more compact than those in group 2 they create more contact regions and more contact pressure on the touching points of adjacent conductive loops. Thus, higher strain rates are needed to separate conductive contact points from each other in tightly knitted structures. Different levels of tightness during the knitting process can cause the different mechanisms to start and finish at different strain levels. When the group 1 sensor in the Figure 5 is considered, the two slopes reflect two different effects. Increasing strains up to 19% cause the upper parts of the limbs of adjacent loops to separate. Above 19%, the conductive loops start to separate from their sinker loops. The mechanism is the same in every case, but different levels of compactness of knitting cause the effects to occur at different levels of strain. When the group 3 samples are considered, it would be appear that there is just one linear range through the whole 40% strain range and the effect has a 0.75 gauge factor value. Group 3 comprises those samples with the most tightly knitted structure and they prove to have the highest linearity with the lowest gauge factor. Table 3 shows the *R*^2^ (coefficient of determination) values of the best fitted linear curves of resistance-strain data of knitted sensors. While the compactness of structure endowed more linearity, the fineness of the elastomeric yarn resulted in the lowest gauge factor value due to the reduced contact area of each of the conductive yarns compared with other samples which have been made using thicker yarns.

**Figure 6.**
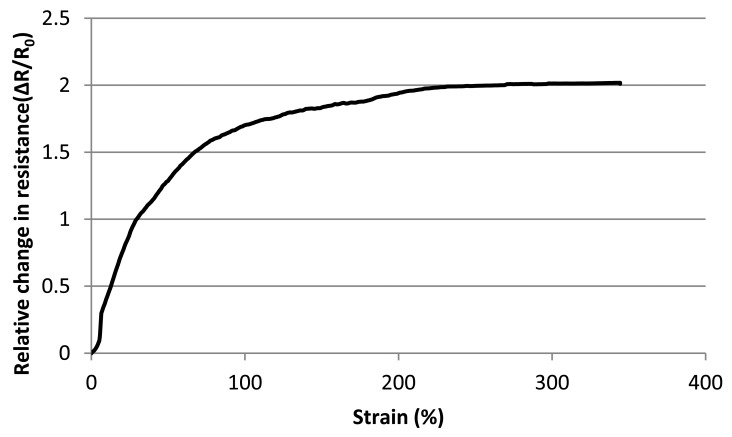
Resistance versus Strain up to breaking point of group 1 sample.

**Figure 7.**
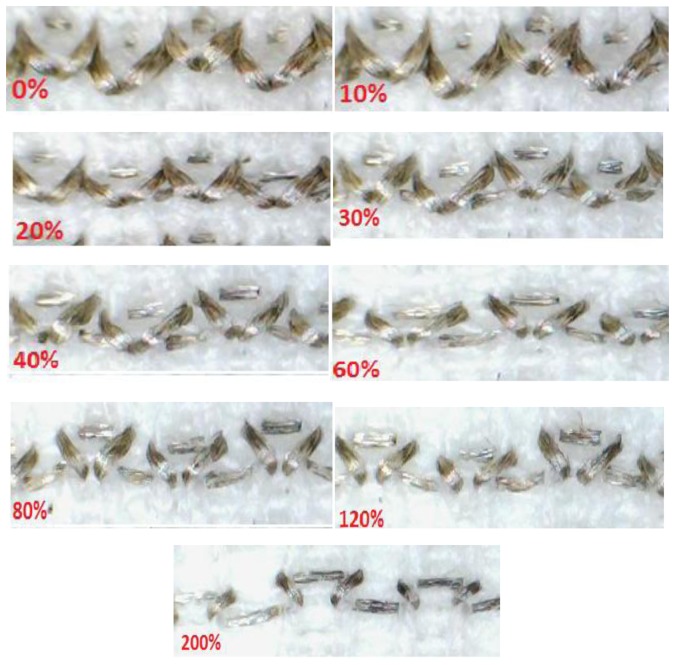
Magnified images of one conductive course during various levels of textile extension from 0% to 200%.

As seen from Figure 6, there is also a contribution to the change of the resistance measurement which derives from strain of the conductive yarn itself. This is felt to be more significant at higher strain rates, as it is at higher strain rates that the knitted loops start to be distorted when the fabric is stretched. Hence, the length of conductive yarn increases and as a result of this, the overall resistance of the sensor continues to increase even though the knitted loops have virtually ceased to make contact at their touching points and Figure 7 shows magnified images of a part of one conductive course during various level of textile extension.

**Figure 8.**
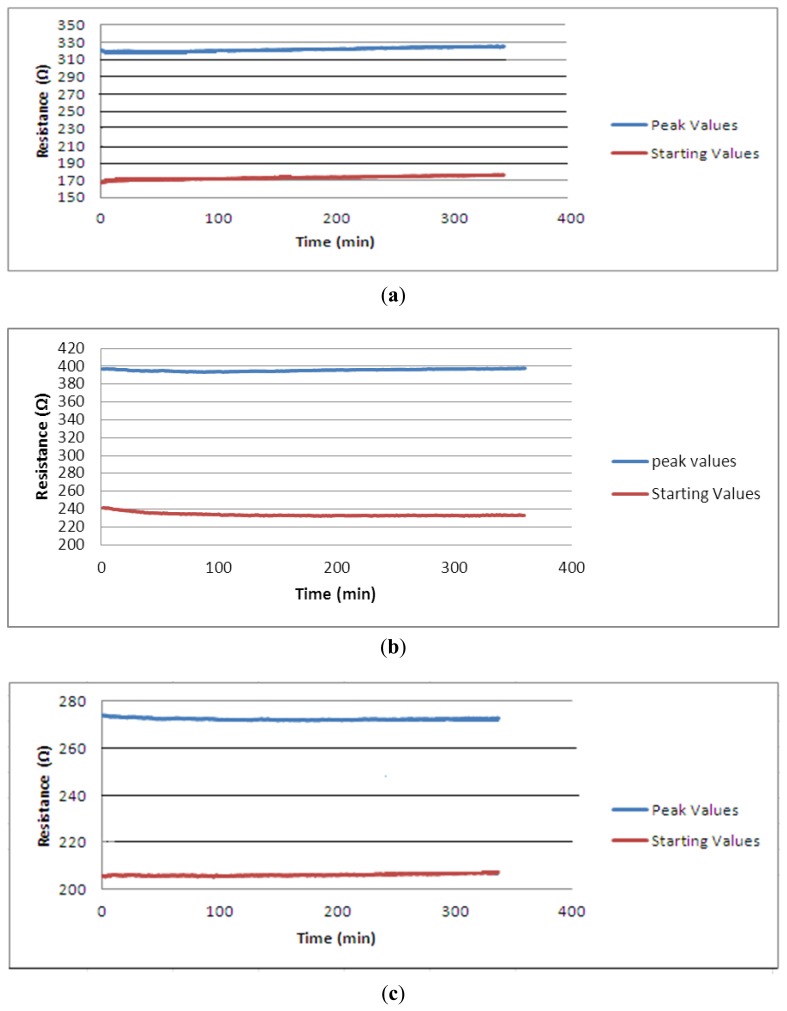
Long term cycling behaviour graphs of three group sensors: (**a**) Group 1; (**b**) Group 2; (**c**) Group 3.

When the sensor is intended to be used for a longer period of time and for more operational cycles proposed sensor needs to show stable properties. Figure 8 shows 1,000 cycle of knitted sensors extended up to 40% strain value. The very small deviation from the horizontal of the top and bottom lines shown in Figure 8 indicate that the sensors are extremely stable.

Over the duration of the test, the unloaded (starting) resistance of the samples increased by just 7 Ω, 6.28 Ω and 2.50 Ω for group 1, group 2 and group 3 sensors, respectively, and the peak (40%) resistance increased by 4 Ω, 0.70 Ω and 2.7 Ω for group 1, group 2 and group 3 sensors, respectively. Thus, the sensor can be considered to be stable over long-term usage. This design of a sensor offers a distinct solution to the drift problem which has been widely reported in textile-based sensors. The base fabric is a modified interlock structure and has been shown to provide particularly stable knitted base. The conductive yarn loops are located onto the technical face of the interlock structure and during tensile testing the applied force gradually separates the fabric wales. Hence, the conductive yarn itself affected minimally by the applied force and it retains its structural properties even longer usage.

**Figure 9.**
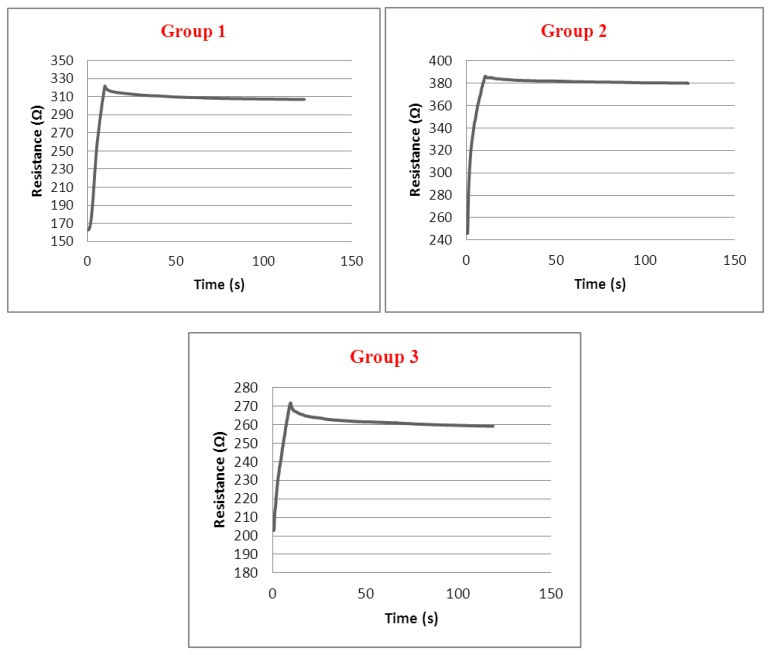
Relaxation behaviour of sensors at 40% strain.

In Figure 9 relaxation behaviour of the sensors has been shown. The dwell times at maximum strains are 2 min. When the strain is kept constant at the 40% strain level, the group 1 sensor relax an average of 14.9 Ω, the group 2 sensors relax by 6.1 Ω and the group 3 sensors relax by 11.4 Ω. These relaxation levels caused inaccuracy of 9.3%, 4.3% and 16.6%, respectively, for each sensor and it seems that when the tightness factor of the structure increases, inaccuracy caused by relaxation increases for a given strain value which is kept equal for each group.

## 4. Conclusions

In this paper, a novel textile-based strain sensor and the effect of base fabric parameters on its sensor properties have been presented. A strong relationship has been established between the sensor characteristics and the base fabric parameters. Variations in elastomeric yarn input tension have greatly affected the sensors' linear range and gauge factor values. More compact structures showed higher linearity in respect of one specific sensor design. Also, the elastomeric yarn linear density affected the contact resistance of the conductive loops by decreasing the number of contact points. The tightness factor values of the base fabric also affected the relaxation behaviour sensors. Less compact structures showed improved accuracy during the relaxation period. The sensor design offers a good solution to the problem of drift which is usually seen in textile- based sensors. Future work includes alternating of conductive yarn input tension and using of different types of conductive yarns in order to see the effect of these variables on the sensor properties.

## Figures and Tables

**Table 1.** Manufacturing parameters of knitted strain-sensors.

**Sample Group**	**Elastomeric Yarn Input Tension (cN/Tex)**	**Elastomeric Yarn Linear Density**	**Effective Conductive Area**	**Number of Conductive Wales**	**Number of Conductive Courses**
**Group 1**	0.125	800	23 mm×42 mm	36	12
**Group 2**	0.062	800	31 mm×44 mm	36	12
**Group 3**	0.125	570	20 mm×41 mm	36	12

**Table 2.** Comparison of average electrical resistance and average base fabric parameters.

**Sample Group**	**Elastomeric Yarn Input Tension (cN/Tex)**	**Elastomeric Yarn Linear Density (dtex)**	**Wale Density (wales per·cm)**	**Course Density (courses per·cm)**	**Structural-Cell Stitch Length (mm)**	**Tightness Factor**	**Electrical Resistance (Ω)**
**Group 1**	0.125	800	8.67	15.55	15.36	1.84	166.47
**Group 2**	0.062	800	8.27	12.20	19.75	1.43	242.02
**Group 3**	0.125	570	8.88	17,15	10.97	2.17	206.58

**Table 3.** *R*^2^ values of knitted sensors.

**Sample Type**	**First Linear Region**	**Second Linear Region**	**Whole working range**
Group 1	0.996	0.997	0.980
Group 2	0.985	0.985	0.868
Group 3	-	-	0.997

## References

[1] Tao X., Tao X. (2005). IntroductionIntroduction. Wearable Electronics and Photonics.

[2] Electrotextiles Conductive materials, textiles. http://openmaterials.org/2011/03/27/materials-101-electrotextiles/.

[3] Kirstein T., Cottet D, Grzyb J, Tröster G., Tao X. (2005). Wearable Computing Sytems—Electronic Textiles. Wearable Electronics and Photonics.

[4] Huang C.T., Tang C.F., Shen C.L. A Wearable Textile for Monitoring Respiration, Using a Yarn-Based Sensor.

[5] Kim K., Lee I.K., Choi S.S., Kim S.S., Lee T.S., Cha E.J. Wearable Transducer to Monitor Respiration in a Wireless Way.

[6] Pacelli M., Loriga G., Paradiso R. Flat knitted sensors for respiration monitoring.

[7] Mitchell E, Coyle S., O'Connor N.E., Diamond D., Ward T. Breathing feedback System with Wearable Textile Sensors.

[8] Guo L., Berglin L., Wiklund U., Mattila H. (2013). Design of a garment-based sensing system for breathing monitoring. Text. Res. J..

[9] Li X., Zhang R, Yu W, Wang K, Wei J, Wu D, Cao A, Li Z, Cheng Y, Zheng Q. (2012). Stretchable and Highly Sensitive Graphene-on-Polymer Strain Sensors; Scientific Reports.

[10] Kannaian T., Neelaveni R., Thilagavathi G. (2011). Development and characterisation of elastomeric tape sensor fabrics for elbow angle measurement. Indian J. Fibre Text. Res..

[11] Scilingo E.P., Lorussi F., Mazzoldi A., De Rossi D. (2003). Strain-sensing fabrics for wearable kinaesthetic-like systems. Sens. J..

[12] Lorussi F., Scilingo E.P., Tesconi A., Tognetti A., De Rossi D. Wearable Sensing Garment for Posture Detection, Rehabilitation and Tele-Medicine.

[13] Xue P., Tao X.M., Kwok K.W., Leung M.Y., Yu T.X. (2004). Electromechanical behavior of fibers coated with an electrically conductive polymer. Text. Res. J..

[14] Mattmann C., Clemens F., Tröster G. (2008). Sensor for measuring strain in textile. Sensors.

[15] Zhang H., Tao X., Wang S., Yu T. (2005). Electro-mechanical properties of knitted fabric made from conductive multi-filament yarn under unidirectional extension. Text. Res. J..

[16] Zhang H., Tao X., Yu T., Wang S. (2006). Conductive knitted fabric as large-strain gauge under high temperature. Sens. Actuat. A: Phys..

[17] Kun Y., Guang-li S., Liang Z., Li-wen L. Modelling the Electrical Property of 1 × 1 Rib Knitted Fabrics Made from Conductive Yarns.

[18] Li L., Au W.M., Li Y., Wan K.M., Wan S.H., Wong K.S. Electromechanical Analysis of Conductive Yarn Knitted in Plain Knitting Stitch under Unidirectional Extension.

[19] Horrocks A.R., Anand S., Anand S.C. (2000). Handbook of Technical Textiles.

[20] Gravas E., Kiekens P., Langenhove L. (2006). Predicting fabric weight per unit area of single and double‐knitted structures using appropriate software. Autex Res. J..

[21] Holm R., Holm E. (1967). Electric Contact Theory and Application.

